# Rho-Kinase Blockade Attenuates Podocyte Apoptosis by Inhibiting the Notch Signaling Pathway in Diabetic Nephropathy

**DOI:** 10.3390/ijms18081795

**Published:** 2017-08-18

**Authors:** Keiichiro Matoba, Daiji Kawanami, Yosuke Nagai, Yusuke Takeda, Tomoyo Akamine, Sho Ishizawa, Yasushi Kanazawa, Tamotsu Yokota, Kazunori Utsunomiya

**Affiliations:** Division of Diabetes, Metabolism and Endocrinology, Department of Internal Medicine, Jikei University School of Medicine, Tokyo 105-8461, Japan, matoba@jikei.ac.jp (K.M.); y.nagai@jikei.ac.jp (Y.N.); ms05-takeda@jikei.ac.jp (Y.T.); akamine-tm@igakuken.or.jp (T.A.); ishizawa@jikei.ac.jp (S.I.); yasu@jikei.ac.jp (Y.K.); yokotat@jikei.ac.jp (T.Y.); kazu-utsunomiya@jikei.ac.jp (K.U.)

**Keywords:** Rho-kinase, Jag1, Notch signaling, diabetic nephropathy

## Abstract

Podocyte apoptosis is a key process in the onset of diabetic nephropathy. A significant body of evidence shows that the Notch signaling pathway plays a central role in this process. We found that Rho-kinase mediates transforming growth factor β (TGF-β)-induced Notch ligand Jag1 expression. Importantly, TGF-β-mediated podocyte apoptosis was attenuated by Rho-kinase inhibition. Mechanistically, Rho-kinase regulated Jag1 induction via the extracellular signal-regulated kinase (ERK) 1/2 and c-Jun N-terminal kinase (JNK) but not Smad pathways. Consistently, the Rho-kinase inhibitor fasudil prevented albuminuria and the urinary excretion of nephrin in *db*/*db* mice and reduced the prevalence of podocyte apoptosis and Jag1 expression. Finally, the expression of Jag1 and apoptosis markers such as Bax and cyclin-dependent kinase inhibitor 1A (CDKN1A) was decreased in podocytes derived from *db*/*db* mice treated with fasudil. The present study provides evidence that Rho-kinase plays a key role in podocyte apoptosis. Rho-kinase is an attractive therapeutic target for diabetic nephropathy.

## 1. Introduction

The number of patients with diabetic nephropathy is increasing worldwide. Diabetic nephropathy is a risk factor not only for end-stage renal disease but also for cardiovascular disease [1]. Therefore, it is of considerable important to establish a novel therapeutic strategy against diabetic renal injury in order to improve the prognosis of these patients as well as relieve the social economic burden. To this end, understanding the molecular mechanisms of diabetic nephropathy is critically important.

Podocytes are highly differentiated epithelial cells and have a limited ability to self-repair and/or regenerate. Therefore, podocyte injury is an important factor in determining the prognosis of diabetic nephropathy. Podocyte foot processes are interconnected by slit diaphragms and form the final filtration barrier.

The slit diaphragm consists of a complex of plasma-membrane proteins, such as nephrin and podocin. Abnormalities of podocyte foot processes, including effacement and apoptosis, are common feature of diabetic nephropathy [2]. Transforming growth factor β (TGF-β) has been implicated as a key regulator of this process [3]. The TGF-β receptor is a serine/threonine kinase transmembrane heteromeric type I and type II receptor complex that delivers signals through Smad family transcription factors upon receptor activation [4]. Importantly, TGF-β also directly activates Smad-independent signaling pathways, such as Rho/Rho-kinase [3] and Notch signaling [5]. Rho-kinase is an effector of the small G protein Rho. Rho-kinase regulates several cellular functions, including cytoskeleton reorganization, proliferation, apoptosis, and gene regulation [6]. We previously reported that Rho-kinase is activated under diabetic conditions and promotes the mesangial production of extracellular matrix by enhancing the inflammatory process [7,8,9,10]. With this background, Rho-kinase inhibitors have been widely used for experimental diabetic nephropathy models. The Rho-kinase inhibitors, fasudil and Y-27632, inhibit Rho-kinase by competing with ATP for the binding site of the kinase catalytic subunit. Fasudil is converted in vivo to its active metabolite hydroxyfasudil which is highly selective for Rho-kinase [11]. Therefore, fasudil is preferentially used for in vivo studies.

Mammalian Notch receptors (Notch 1–4) are a family of transmembrane proteins. Notch signaling is short-distanced, as it requires cell–cell contact to be initiated [5]. Notch receptors consist of two domains, namely the N-terminal Notch extracellular domain (NECD) and the C-terminal Notch intracellular domain (NICD). There are two families of Notch ligands: Jagged (Jag 1–2) or Δ-like (Dll 1–4). Upon the binding of Notch receptors to Notch ligands, NICD is released from the cell membrane by γ-secretase and translocates to the nucleus. The released NICD then forms a complex with the recombination signal binding protein for immunoglobulin κJ region (Rbpj) and mastermind-like (MAM) proteins to initiate target gene transcription [12,13]. Notch signaling has been implicated as a critical regulator of the cell fate during kidney development, including nephron segmentation and endowment [14,15,16,17]. However, Notch ligands and receptors are not present in mature podocytes and are not required for podocyte development [14,18,19,20].

Interestingly, a significant body of evidence has shown that Notch signaling is induced under diabetic conditions. Niranjan et al. demonstrated that renal Notch signaling is activated in both type 1 and type 2 diabetic mice and promotes albuminuria [21]. Furthermore, TGF-β has been shown to increase the Notch1 receptor and Jag1 expression in podocytes [21,22], which is associated with cell apoptosis.

In this study, we investigated the involvement of Rho-kinase in Notch signaling-mediated podocyte apoptosis under diabetic conditions.

## 2. Results

### 2.1. TGF-β Induces Jag1 Expression and Rho/Rho-Kinase Activation in Podocytes

**Figure 1:**
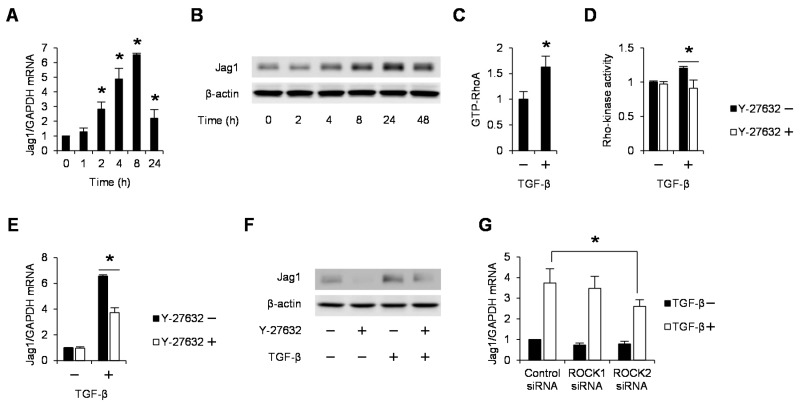
Rho-Kinase mediates transforming growth factor β (TGF-β)-induced Jag1 expression in podocytes. Differentiated E11 podocytes were stimulated with TGF-β (5 ng/mL) for the indicated durations (**A,B**). (**A**) RNA was extracted, and Jag1 mRNA was analyzed by real-time quantitative PCR, with GAPDH mRNA as the internal standard; (**B**) The amount of Jag1 protein in cell lysates from podocytes was determined by Western blotting; (**C**) Cell lysates were collected from E11 podocytes stimulated with TGF-β (5 ng/mL) for 1 min. RhoA activity was determined by G-LISA RhoA assay; (**D**) Podocytes were pretreated with Y-27632 (10 µM) and then stimulated with TGF-β (5 ng/mL) for 1 h. Rho-kinase activity was measured as described in the Materials and methods section; (**E**) E11 podocytes were pretreated with Y-27632 (10 µM) before stimulation with TGF-β (5 ng/mL) for 8 h. Jag1 mRNA was analyzed by real-time quantitative PCR; (**F**) Podocytes were stimulated by TGF-β for 24 h after treatment with Y-27632. The protein expression of Jag1 was analyzed by Western blotting. A representative blot of three independent experiments is shown; (**G**) Podocytes stimulated with TGF-β (8 h) were treated with scramble control siRNA or Rho-kinase isoform specific siRNA and analyzed by real-time quantitative PCR. * *p* < 0.05 vs. control siRNA with TGF-β. The data are presented as means ± SD.

Notch ligand Jag1 was induced by the treatment of recombinant TGF-β at both the mRNA (Figure 1A) and protein (Figure 1B) levels in murine podocytes E11. Similarly, TGF-β activated RhoA (Figure 1C) and Rho-kinase, which was completely abolished with the pretreatment with the specific Rho-kinase inhibitor Y-27632 (Figure 1D). Real-time quantitative PCR (Figure 1E) and Western blot analyses (Figure 1F) showed the inhibitory effects of Y-27632 on the TGF-β-mediated Jag1 expression, indicating the participation of Rho-kinase in Jag1 induction. To elucidate the isoform-specific roles of Rho-kinase, we knocked down ROCK1 and ROCK2 separately using siRNA duplexes (Figure 1G). Individual knockdown of ROCK2 but not ROCK1 attenuated Jag1 induction. These data indicate that TGF-β promotes Jag1 production via a ROCK2-dependent pathway in podocytes.

### 2.2. Rho-Kinase Inhibition Attenuates Podocyte Apoptosis

**Figure 2:**
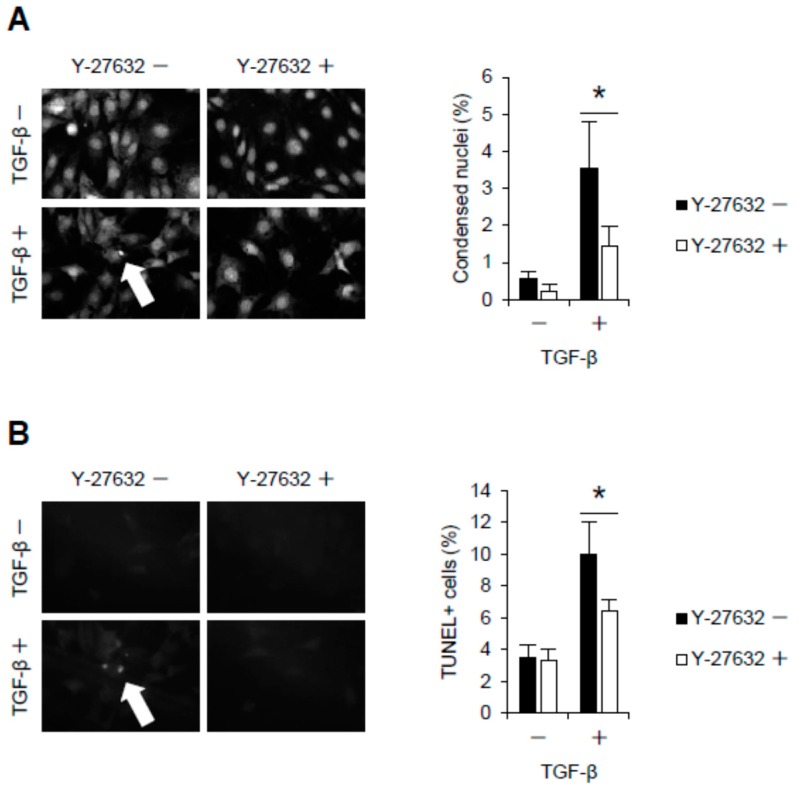
Rho-Kinase mediates podocyte apoptosis. (**A**) Representative photomicrographs and quantification of condensed nuclei in podocytes. E11 podocytes were pretreated with Y-27632 (10 µM) and then stimulated with TGF-β (5 ng/mL) for 24 h; (**B**) Microphotographs and quantification of TUNEL-positive apoptotic podocytes. Original magnification, ×400. * *p* < 0.05. The data are presented as means ± SD.

We next investigated the involvement of TGF-β-mediated Jag1 signaling in apoptosis of podocytes. To evaluate apoptosis, condensed nuclei and TUNEL-positive cells were counted among podocytes under TGF-β-stimulated conditions. While the number of condensed nuclei was increased in TGF-β-treated podocytes, this effect was reversed when the cells were pretreated with the Rho-kinase inhibitor Y-27632 (Figure 2A). TUNEL staining also showed increased apoptosis in podocytes treated with TGF-β (Figure 2B), and this increase was suppressed by Rho-kinase inhibition.

### 2.3. Rho-Kinase Promotes TGF-β-Induced Apoptosis in Podocytes via the Notch and Mitogen-activated Protein Kinase (MAPK) Signaling Pathways Independently of the Smads Cascade

**Figure 3:**
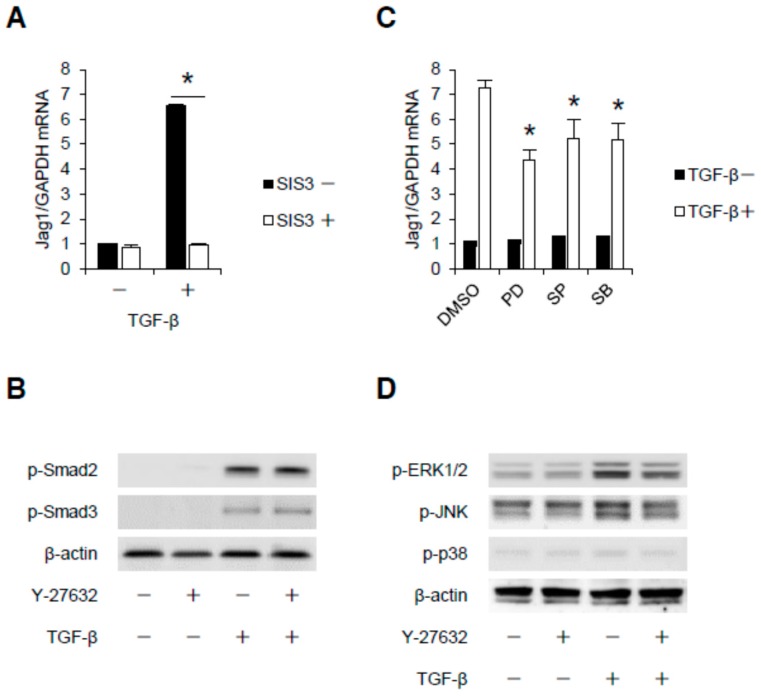
Mitogen-activated protein kinases (MAPKs) are signaling intermediates required for Rho-kinase-mediated Jag1 induction. (**A**) Differentiated E11 podocytes were pretreated with Smad3 inhibitor (SIS3, 10 µM) and then stimulated with TGF-β (5 ng/mL) for 8 h. Jag1 mRNA was analyzed by real-time quantitative PCR; (**B**) Podocytes were pretreated with Y-27632 (10 µM) before stimulation with TGF-β (5 ng/mL) for 30 min. Cell lysates were subjected to Western blotting. A representative blot of three independent experiments is shown; (**C**) Podocytes were stimulated with TGF-β for 8 h with or without pretreatment of MAPK inhibitors (50 µM). RNA was extracted, and Jag1 mRNA was analyzed by real-time quantitative PCR, with GAPDH mRNA as the internal standard. * *p* < 0.05 vs. DMSO with TGF-β; (**D**) Podocytes were pretreated with Y-27632 (10 µM) and then stimulated with TGF-β (5 ng/mL) for 30 min. Equal amounts of cell lysate were subjected to Western blotting using MAPKs antibodies. A representative blot of three independent experiments is shown. The data are presented as means ± SD.

We next addressed the contribution of Rho-kinase to the TGF-β-induced Smad cascade. SIS3, a selective inhibitor of Smad3, completely abolished Jag1 induction (Figure 3A), indicating the involvement of Smad signaling in Jag1 transcription in podocytes. The activation of Smad signaling by TGF-β was also confirmed by investigating the phosphorylation of Smads 2 and 3 (Figure 3B). However, the TGF-β-mediated phosphorylation of Smads 2 and 3 was not affected by Y-27632. Jag1 induction is also mediated by MAPK pathways, as evidenced by the effects of pharmacological inhibitors for mitogen-activated protein/extracellular signal-regulated kinase kinase (MEK) 1/2, c-Jun N-terminal kinase (JNK) or p38 MAPK (Figure 3C). As shown in Figure 3D, the TGF-β-induced phosphorylation of extracellular signal-regulated kinase (ERK) 1/2 and JNK was attenuated by Rho-kinase blockade. These data indicate that ERK1/2 and JNK are critical signaling intermediates required for Rho-kinase-mediated Jag1 induction in podocytes, independently of the Smads cascade.

### 2.4. Rho-Kinase Inhibitor Fasudil Inhibits Podocyte Apoptosis in db/db Mice

**Figure 4:**
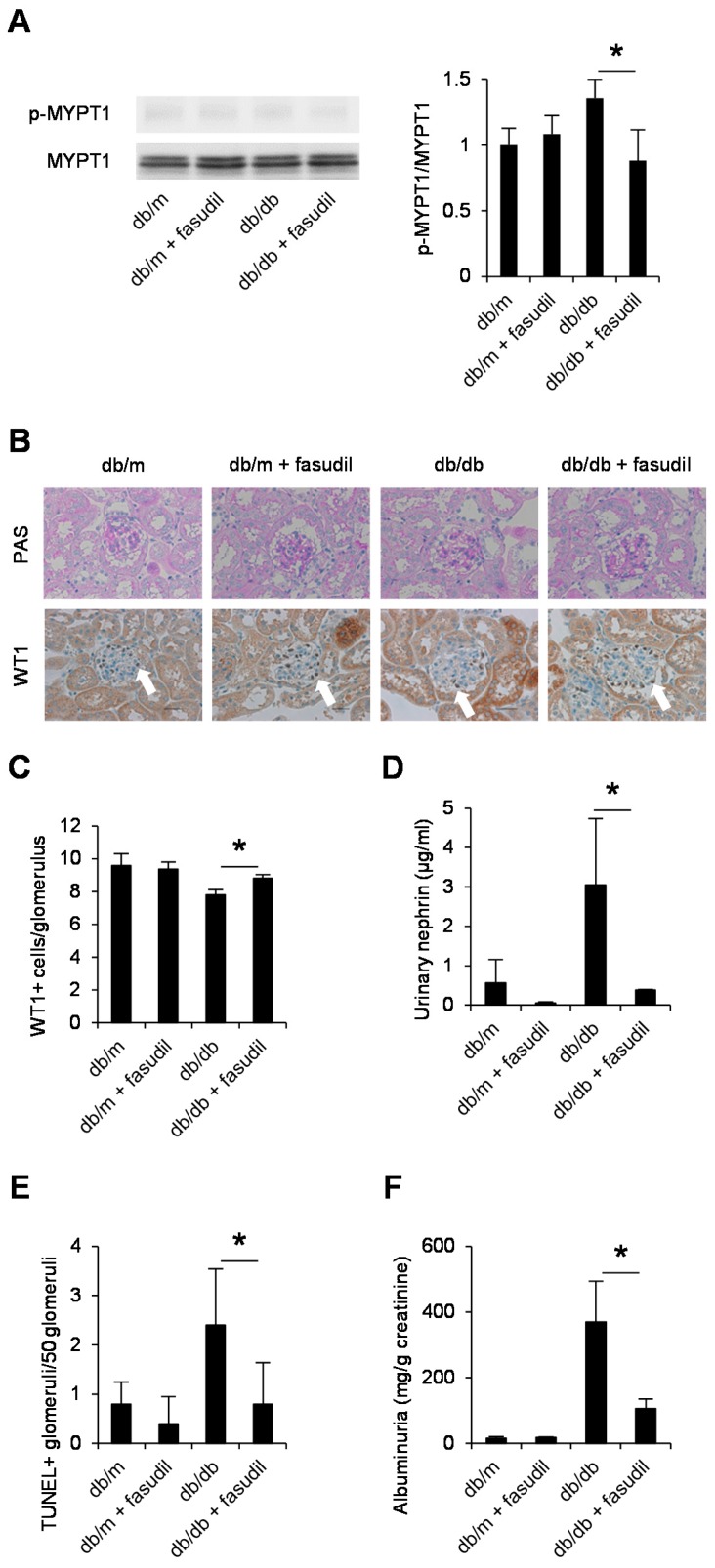

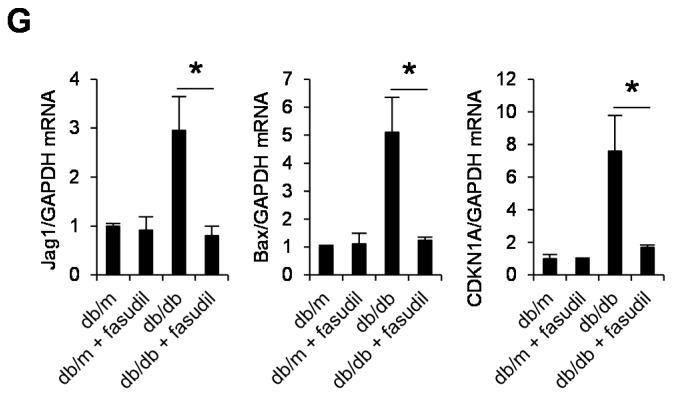
Treatment of *db*/*db* mice with fasudil prevents podocyte loss. (**A**) A representative blot of three independent experiments and quantitative analyses of the phosphorylated form of myosin phosphatase target subunit 1 (MYPT1) (p-MYPT1) and MYPT1 in protein extracts from the renal cortex of *db*/*m* mice, *db*/*m* mice given fasudil (*db*/*m* + fasudil), *db*/*db* mice, and fasudil-treated *db*/*db* mice (*db*/*db* + fasudil); (**B**) Representative photomicrographs of periodic acid-Schiff (PAS) and WT1-stained kidney sections. Original magnification, ×400; Quantitative analyses of WT1-positive cells in (**C**) the glomerulus and the urinary excretion of nephrin; (**D**–**F**) TUNEL-stained glomerular cells and changes in the albumin excretion in each group; (**G**) Jag1 and apoptotic markers were analyzed in the isolated podocytes. * *p* < 0.05. The data are presented as the means ± SD. (*n* = 5 in each group).

To clarify the involvement of Rho-kinase in apoptosis of podocytes and the progression of diabetic nephropathy in vivo, we next assessed the effect of Rho-kinase blockade on podocyte loss and albuminuria using *db*/*db* mice as a model of type 2 diabetes. Many studies, including our own, have shown that the up-regulation of TGF-β in the renal cortex plays a critical role in the development of diabetic nephropathy in these mice [8]. Drug treatment was started from five weeks of age, and mice were sacrificed at eight weeks of age, when they began to show a marked increase in podocyte apoptosis [23]. We first confirmed activated Rho-kinase in the renal cortex of *db*/*db* mice by the quantification of phosphorylation of MYPT1 (myosin phosphatase target subunit 1), a substrate of Rho-kinase, with a reduction in its levels by fasudil treatment (Figure 4A). The glomerular structure in *db*/*db* mice showed no abnormalities at eight weeks of age (Figure 4B); however, these mice showed decreased numbers of glomerular podocytes (Figure 4B,C) with an increased excretion of urinary nephrin (Figure 4D). Importantly, the administration of fasudil prevented podocyte loss (Figure 4C,D), increases in TUNEL-positive glomerular cells (Figure 4E), and albuminuria (Figure 4F) in diabetic mice. We then investigated the effects of Rho-kinase inhibition on podocytes isolated from the same mice. As shown in Figure 4G, increased Jag1 expression levels were observed in isolated podocytes derived from *db*/*db* mice, and this increase was inhibited by fasudil. A similar trend was observed in the expression of Bax and cyclin-dependent kinase inhibitor 1A (CDKN1A). Taken together, these data demonstrate the critical role of Rho-kinase in Jag1-mediated apoptotic events in vivo.

## 3. Discussion

Glomerular podocytes serve as an essential component of the filtration barrier. It is important to delineate the processes that mediate podocyte damage. Using functional, biochemical, and genetic approaches, we identified Rho-kinase as a critical regulator of Notch signaling. Our data provide evidence that Rho-kinase plays a central role in podocyte apoptosis, which subsequently influences albuminuria.

Smad proteins are divided into three groups: receptor-activated type (R-Smads: Smads 1–3, 5 and 8), co-mediator type (Smads 4 and 10), and inhibitory type (Smads 6 and 7). Among R-Smads, Smads 2 and 3 are involved in TGF-β-mediated Notch activation in the kidney [24]. However, Rho-kinase did not affect the Smads 2 and 3 phosphorylation in podocytes in this study. This finding is consistent with those of previous reports showing that Rho-kinase inhibition has no effect on Smads 2 and 3 phosphorylation in mesangial cells [25]. We therefore examined the contribution of Rho-kinase to Smad-independent pathways. TGF-β is known to activate the ERK, JNK, and p38 MAPK pathways independently of the Smads cascade [3]. Of these, phosphorylation of ERK and JNK was attenuated by a Rho-kinase inhibitor, indicating that ERK/JNK lies downstream of Rho-kinase. ERK, JNK, and p38 MAPK seem to commonly play important roles in podocyte apoptosis, regardless of stimuli, as seen in angiotensin II (AngII)-induced podocyte apoptosis [26]. Previous studies have shown that ERK mediates TGF-β-induced apoptosis in podocytes by increasing oxidative stress and inflammation via mTOR and NF-κB activation [27,28]. TGF-β induces biphasic ERK phosphorylation in podocytes [27]. The rapid phase (starting at 5 min after TGF-β stimulation) occurs in a Smad-independent fashion [29], while the late phase (starting at 6 h after TGF-β stimulation) occurs in a Smad-dependent fashion [30]. As Rho-kinase is not involved in the Smad-dependent signaling pathway, it is likely that Rho-kinase mediates the Smad-independent ERK phosphorylation. JNK also plays an important role in AngII-induced podocyte apoptosis [26]. In the present study, we showed that JNK mediates TGF-β-induced podocyte apoptosis. Although p38 MAPK was involved in the podocyte apoptosis induced by TGF-β, we failed to show the contribution of Rho-kinase to p38 MAPK activation, whereas Rho-kinase inhibition attenuated the TNF-α-induced p38MAPK activation in mesangial cells [7]. These observations indicate that MAPKs mediate Rho-kinase-dependent pathways, but each MAPK is regulated distinctively with different stimuli and cell types.

The Notch pathway has been shown to interact with the TGF-β pathway in podocyte apoptosis. These pathways form a positive feedback loop, as Jag1 and TGF-β upregulate each other [31]. Furthermore, TGF-β has been shown to upregulate TGF-β in the kidney of diabetic mice in a process that has been proposed to be positive feedback keeping the TGF-β signal active under conditions of diabetic nephropathy [32,33]. We previously reported that Rho-kinase inhibition attenuates the TGF-β expression in the renal cortex of *db*/*db* mice [8]. Given the ability of Rho-kinase inhibitors to attenuate both TGF-β and TGF-β-mediated Jag1 expression, Rho-kinase may be a regulator of the positive feedback loop formed by TGF-β and Notch signaling. In the present study, we mainly focused on the mechanisms by which Rho-kinase regulates TGF-β-induced Notch signaling activation and subsequent podocyte apoptosis. Further studies to elucidate how Rho-kinase regulates these complicated positive feedback loops will be required.

Rho-kinase has two isoforms: ROCK1 and ROCK2. In this study, ROCK2 but not ROCK1 was dominantly involved in the TGF-β-mediated Jag1 expression. These isoforms share 65% identity overall in their amino acid sequences and 92% identity in their kinase domains [34], and each has its own specific functions. For instance, it has been shown that ROCK2 but not ROCK1 is involved in NF-κB-mediated induction of cell adhesion molecule [35] and in VEGF-driven angiogenesis [36]. In contrast, ROCK1 but not ROCK2 mediates leukocyte recruitment and neointima formation following vascular injury [37]. ROCK1 and ROCK2 possess absolute identity in the ATP-binding pocket. Since Y-27632 and fasudil target their ATP-dependent kinase domains, these drugs inhibit ROCK1 and ROCK2 at equimolar concentrations. We previously reported that ROCK1 and ROCK2 equally contributed to hypoxia-inducible factor 1α (HIF-1α) stabilization in mesangial cells [8]. How ROCK2 dominantly regulates Jag1 expression in podocytes is unclear at present. We speculate that the ROCK isoforms have distinct functions in each type of cell in the glomerulus. Wang et al. showed that the podocyte-specific overexpression of ROCK1 promotes glomerulosclerosis by increasing mitochondrial fission [38]. They also showed that podocyte effacement was induced by ROCK1 overexpression. Further studies using cell-specific gene deletion approaches in animal models will be required to elucidate the distinct roles of ROCK1 and ROCK2 in podocytes.

## 4. Materials and Methods

### 4.1. Reagents

Recombinant murine TGF-β was purchased from R&D Systems (Minneapolis, MI, USA). Y-27632 (Rho-kinase inhibitor) was obtained from Wako (Tokyo, Japan). Fasudil was kindly provided by Asahi Kasei Pharma Corporation (Tokyo, Japan). Predesigned murine siRNA duplexes were purchased from Santa Cruz Biotechnology (Dallas, TX, USA). PD98059 (MEK1/2 inhibitor), SP600125 (JNK inhibitor), and SB203580 (p38 MAPK inhibitor) were from Tocris Biosciences (Bristol, UK). Antibodies of Jag1, phospho-Smad2 (Ser465/467), phospho-Smad3 (Ser423/425), phospho-ERK1/2 (Thr202/204), phospho-JNK (Thr183/Try185), phospho-p38 MAPK (Thr180/Tyr182), and myosin phosphatase target subunit 1 (MYPT1) were purchased from Cell Signaling Technology (Danvers, MA, USA). β-Actin antibody and horseradish peroxidase-conjugated antibody were from Santa Cruz Biotechnology. Phospho-MYPT1 antibody (Thr850) was obtained from Millipore (Billerica, MA, USA).

### 4.2. Cell Culture

A conditionally immortalized murine podocyte cell line (E11) was obtained from Cell Line Services. Podocytes were propagated at 33 °C in RPMI1640 supplemented with 10% FBS and 10 U/mL of IFN-γ (Saint Louis, MO, USA) to enhance expression of thermosensitive T antigen. To induce differentiation, podocytes were maintained at 37 °C without IFN-γ for 10–14 days. For experiments with pharmacological inhibitors, podocytes were incubated with the indicated concentration of agents for 1 h before exposure to TGF-β.

### 4.3. Animal Studies

Five-week-old male *db*/*db* mice and their age-matched heterozygous male littermates *db*/*m* mice were obtained from CLEA Japan, Inc. (Tokyo, Japan) and housed in an animal facility with a 12-h light-dark cycle and unlimited access to food and water for the duration of the study. These mice were divided into the following groups: non-diabetic *db*/*m* mice (*n* = 5), *db*/*m* mice given fasudil (*n* = 5), type 2 diabetic *db*/*db* mice (*n* = 5), and *db*/*db* mice treated with Rho-kinase inhibitor fasudil (*n* = 5). Fasudil was administered in the drinking water (100 mg/kg/day). The kidney was perfused with ice-cold phosphate-buffered saline (PBS) and rapidly dissected. All procedures were conducted in accordance with institutional guidelines for the care and use of laboratory animals at Jikei University School of Medicine. (#22-002, 2 June 2010).

### 4.4. Podocyte Isolation

Podocyte isolation was performed as described previously [39]. After red blood cell removal using ammonium chloride potassium lysis buffer and endothelial cell depletion using CD31 antibody (Biolegend, San Diego, CA, USA), nephrin-positive cells were prepared from minced murine kidneys using magnet-activated cell sorting with nephrin antibody (Thermo Fisher Scientific, Waltham, MA, USA).

### 4.5. Silencing of Rho-Kinase

Podocytes were transfected with control siRNA (negative control) or siRNA (50 nM) against ROCK1 or ROCK2 using Lipofectamine reagent (Invitrogen, Carlsbad, CA, USA) in accordance with the manufacturer’s instructions.

### 4.6. RNA Isolation and Real-Time Quantitative Polymerase Chain Reaction

Total RNA was isolated from podocytes with TRIzol reagent (Invitrogen) followed by chloroform-isopropanol extraction and ethanol precipitation, and 1 µg of total RNA was reverse-transcribed using the Prime Script RT reagent Kit (Takara Bio, Kusatsu, Japan). To evaluate the mRNA expression of Jag1, real-time quantitative polymerase chain reaction (PCR) was performed using the Thermal Cycler Dice Real Time System TP800 (Takara Bio) with SYBR Green I fluorescence signals. The levels of Jag1 mRNA were normalized with GAPDH and expressed as levels relative to control.

### 4.7. Western Blot Analyses

Podocytes were lysed with RIPA buffer. Equal amounts of protein samples were subjected to Western blot as described previously [9]. Immunoreactive bands were visualized with the enhanced chemiluminescence system (Amersham, GE Healthcare Life Science, Marlborough, MA, USA). The peroxidase luminescence intensity was measured using an LAS-4000 mini Luminescent Image Analyzer (FUJIFILM, Tokyo, Japan). Quantification was performed in three independent experiments.

### 4.8. GTP-RhoA Activity Assay

Activation of RhoA was measured by the quantification of guanosine-5’-triphosphate (GTP)-bound RhoA using a G-LISA RhoA activation kit in accordance with the manufacturer’s instructions (Cytoskeleton, Denver, CO, USA). Briefly, the collected podocytes were immediately snap-frozen to prevent RhoA degradation and then lysed in ice-cold lysis buffer. The protein samples were added to a 96-well microplate coated with Rho-GTP binding protein. The plates were then incubated with RhoA antibody and secondary horseradish peroxidase-conjugated antibody. The luminescence signal was detected by measuring the absorbance at 490 nm.

### 4.9. Rho-Kinase Activity Assay

The in vitro Rho-kinase activity was determined using a Rho-kinase activity assay kit (Cell Biolabs, San Diego, CA, USA). This kit is designed as an enzyme immunoassay for the specific detection of the phosphorylated form of MYPT1 at Thr696 by Rho-kinase. A microtiter plate is precoated with recombinant MYPT1 protein. After the wells are incubated with cell lysates, phosphorylated MYPT1 is detected by an anti-phospho-MYPT1 (Thr696) antibody followed by incubation with the antibody detection reagent.

### 4.10. Detection of Condensed Nuclei and Apoptotic Podocytes

For the detection of condensed nuclei, podocytes were incubated with propidium iodide. Images were observed on an IX70 microscope (Olympus, Tokyo, Japan) using the MetaVue imaging software program (Molecular Devices, Sunnyvale, CA, USA). Apoptotic podocytes were visualized by the terminal deoxynucleotidyl transferase-mediated deoxyuridine triphosphate-biotin nick end labeling (TUNEL) method using an in situ apoptosis detection kit (Takara Bio) in accordance with the manufacturer’s protocol.

### 4.11. Statistical Analyses

Data are expressed as mean ± standard deviation. The comparison of groups was performed using an analysis of variance and Bonferroni post hoc correction. A value of *p* < 0.05 was considered statistically significant.

## 5. Conclusions

Our findings provide evidence that Rho-kinase blockade inhibits diabetic nephropathy by attenuating podocyte apoptosis. Rho-kinase may be an attractive therapeutic target against diabetic nephropathy.
